# Development and validation of a predictive model for prolonged length of stay in elderly type 2 diabetes mellitus patients combined with cerebral infarction

**DOI:** 10.3389/fneur.2024.1405096

**Published:** 2024-08-01

**Authors:** Mingshan Tang, Yan Zhao, Jing Xiao, Side Jiang, Juntao Tan, Qian Xu, Chengde Pan, Jie Wang

**Affiliations:** ^1^Department of Neurology, Affiliated Banan Hospital of Chongqing Medical University, Chongqing, China; ^2^Operation Management Office, Affiliated Banan Hospital of Chongqing Medical University, Chongqing, China; ^3^Library, Chongqing Medical University, Chongqing, China

**Keywords:** type 2 diabetes mellitus, cerebral infarction, length of stay, prediction model, nomogram

## Abstract

**Background:**

This study aimed to identify the predictive factors for prolonged length of stay (LOS) in elderly type 2 diabetes mellitus (T2DM) patients suffering from cerebral infarction (CI) and construct a predictive model to effectively utilize hospital resources.

**Methods:**

Clinical data were retrospectively collected from T2DM patients suffering from CI aged ≥65 years who were admitted to five tertiary hospitals in Southwest China. The least absolute shrinkage and selection operator (LASSO) regression model and multivariable logistic regression analysis were conducted to identify the independent predictors of prolonged LOS. A nomogram was constructed to visualize the model. The discrimination, calibration, and clinical practicality of the model were evaluated according to the area under the receiver operating characteristic curve (AUROC), calibration curve, decision curve analysis (DCA), and clinical impact curve (CIC).

**Results:**

A total of 13,361 patients were included, comprising 6,023, 2,582, and 4,756 patients in the training, internal validation, and external validation sets, respectively. The results revealed that the ACCI score, OP, PI, analgesics use, antibiotics use, psychotropic drug use, insurance type, and ALB were independent predictors for prolonged LOS. The eight-predictor LASSO logistic regression displayed high prediction ability, with an AUROC of 0.725 (95% confidence interval [CI]: 0.710–0.739), a sensitivity of 0.662 (95% CI: 0.639–0.686), and a specificity of 0.675 (95% CI: 0.661–0.689). The calibration curve (bootstraps = 1,000) showed good calibration. In addition, the DCA and CIC also indicated good clinical practicality. An operation interface on a web page (https://xxmyyz.shinyapps.io/prolonged_los1/) was also established to facilitate clinical use.

**Conclusion:**

The developed model can predict the risk of prolonged LOS in elderly T2DM patients diagnosed with CI, enabling clinicians to optimize bed management.

## 1 Introduction

A bi-directional link has been reported between type 2 diabetes mellitus (T2DM) and cerebral infarction (CI), with their coexistence significantly increasing the incidence rate and mortality (1). Cerebrovascular disease is a common complication in the elderly diabetic population (2), and CI in elderly diabetes patients accounts for 89.1% of all elderly diabetes cerebrovascular disease patients (3, 4). Diabetes is also an independent risk factor for CI. A previous study reported that diabetic patients with cardiovascular and cerebrovascular diseases were at a 2–5 times higher risk than the general population (5). In addition, patients with diabetes combined with CI are more likely to exhibit vascular wall degeneration, necrosis, and functional damage of the fibrinolysis system (6, 7). The condition seriously affects the ability of patients to take care of themselves, greatly reduces the quality of life of patients, and poses a medical burden on society (8).

As one of the factors contributing to total hospitalization costs, prolonged length of stay (LOS) is highly predictive of inpatient costs (9–11). Since LOS is the main determinant of care and rehabilitation costs for elderly T2DM patients diagnosed with CI, determining independent predictors of LOS is crucial to optimizing resource allocation and cost efficiency (12). Furthermore, patients with CI having prolonged LOS are more likely to develop complications such as hypertension or chronic lung disease (13). Prolonged LOS is also associated with increased depression and disability, which negatively impact the quality of life of patients (14). Therefore, understanding the factors related to LOS and estimating the risk of prolonged LOS can predict the recovery after CI, which has great clinical significance.

At present, relatively few studies have investigated the risk of LOS for elderly T2DM patients combined with CI. Therefore, this study aimed to identify potential risk factors for prolonged LOS in elderly T2DM patients combined with CI and construct a predictive model, providing a quick, accurate, and dynamic evaluation method of the risk of prolonged LOS in patients. Consequently, high-risk populations can be managed early, thereby guiding clinical management and nursing.

## 2 Methods

### 2.1 Study design and patients

In this retrospective and multicenter study, the electronic medical records (EMRs) data of 13,361 elderly T2DM patients diagnosed with CI were obtained from five tertiary hospitals in southwest China from 2012 to 2023. A total of 827 patients were recruited from Chongqing Southeast Hospital, 4,763 from the Second Affiliated Hospital of Chongqing Medical University, 1,432 from the University-Town Hospital of Chongqing Medical University, and 1,583 from the Third Affiliated Hospital of Chongqing Medical University. Patients from these four hospitals were divided randomly (7:3) into the training set (*n* = 6,023) and the internal validation set (*n* = 2,582). Patients recruited from the Affiliated Banan Hospital of Chongqing Medical University were used as the external validation set (*n* = 4,756). Supplementary Table S1 displays the data from the five hospitals.

This study was approved by the Ethics Committee of the Affiliated Banan Hospital of Chongqing Medical University (Ethical approval No. BNLLKY2023037) and was conducted in accordance with the Declaration of Helsinki and Good Clinical Practice guidelines. Informed consent for participation was not required due to its retrospective design, and the study was conducted in accordance with national legislation and institutional requirements.

### 2.2 Inclusion and exclusion criteria

The inclusion criteria were as follows: (i) age ≥ 65 years, (ii) data obtained from 2012 to 2023 (iii) hospitalization(s) for T2DM complicated with CI. The exclusion criteria were: (i) LOS < 2 days, (ii) death during hospitalization, and (iii) discharge against medical advice. The selection process is illustrated in Supplementary Figure S1.

### 2.3 Definition

LOS was calculated from the date of admission to the date of checkout. An LOS exceeding the third quartile value of the study population was defined as prolonged LOS (15–17). Specifically, hospitalization <16 days was defined as normal LOS; whereas, hospitalization ≥16 days was defined as prolonged LOS.

### 2.4 Data collection

A total of 36 candidate variables were selected to identify a prolonged LOS. Specifically, this study explored age, sex, insurance type, marriage, past surgical history (PSH), past medical history (PMH), smoking history, drinking history, age-adjusted Charlson comorbidity index (ACCI) score, hypertension, coronary heart disease (CHD), osteoporosis (OP), hyperlipidemia, heart failure (HF), pulmonary infection (PI), analgesics use, antibiotics use, psychotropic drug use, systolic blood pressure (SBP), diastolic blood pressure (DBP), total bilirubin (TBIL), alkaline phosphatase (AKP), direct bilirubin (DBIL), aspartate aminotransferase (AST), alanine aminotransferase (ALT), total cholesterol (TC), triglycerides (TGs), neutrophil-lymphocyte ratio (NLR), lymphocyte-monocyte ratio (LMR), platelet-lymphocyte ratio (PLR), creatinine (CREA), uric acid (UA), low-density lipoprotein cholesterol (LDL-C), high-density lipoprotein cholesterol (HDL-C), albumin (ALB), and fasting blood glucose (FBG).

In this study, the insurance type was divided into the following four categories: urban employee medical insurance (UEMI), urban resident medical insurance (URMI), fully self-paid, and other insurance. Marriage status was also categorized into married, unmarried, and divorced & separated & widowed (DSW).

### 2.5 Statistical analyses

Statistical analyses were performed using SPSS 22.0 and R (version 4.0.2, Vienna, Austria). Continuous variables conforming to a normal distribution were described using mean ± standard deviation, and an independent sample t-test was used for inter-group comparison. Variables not conforming to a normal distribution were described using the median and interquartile range [M(Q25–Q75)] and between-group comparisons were performed using the Mann–Whitney U test. The enumerative data were expressed by frequency and percentage, and the chi-square test was used for comparisons between groups. Missing data were replaced using multiple imputations, which were performed using chained equations and predictive mean matching. In addition, least absolute shrinkage and selection operator (LASSO) regression and multivariable logistic regression analyses were conducted to identify the independent predictors. On the one hand, LASSO has great advantages in selecting explanatory variables, processing high-dimensional data, and solving multicollinearity problems. On the other hand, Lasso regression is also an efficient model that both shrinks and selects regression coefficients and allows for better interpretability of the model (18).

Firstly, the receiver operating characteristic (ROC) curve was plotted to describe the discriminative ability of the model. The closer the ROC curve is to the upper left corner, the higher the sensitivity and specificity of the model. Subsequently, the area under the ROC curve (AUROC) was calculated to quantitatively evaluate the discriminative ability. Moreover, calibration curves were used to evaluate the accuracy of the model’s predicted probability, with the probability curve being closer to the actual probability curve indicating a better fit. Finally, decision curve analysis (DCA) and clinical impact curves (CIC) were performed on the model to evaluate its practicality and benefits in clinical applications by calculating the benefits and losses of patients under different thresholds. In this study, *p* < 0.05 was considered statistically significant.

## 3 Results

### 3.1 Patient characteristics

A total of 13,361 patients were included in the study, with 6,023, 2,582, and 4,756 patients in the training, internal validation, and external validation sets, respectively. In the training and internal validation sets, the median age was 76.00 years (IQR: 71.00, 82.00), and 53.41% were female. 75.61% of patients’ insurance schemes were UEMI. In addition, 62.82% of patients had PSH, 97.34% of patients had PMH, and 16.58% of patients also suffered from hyperlipidemia (Table 1). The Mann–Whitney U test revealed no significant difference in all missing variables before and after multiple imputations (Supplementary Tables S2, S3).

**Table 1:** Baseline characteristics of elderly T2DM patients complicated with CI.

Variables	Total (*N* = 8,605)	Training set (*N* = 6,023)	Internal validation set (*N* = 2,582)	*p* values
LOS	11.00 (7.00,16.00)	11.00 (7.00,16.00)	11.00 (7.00,16.00)	0.484
Age	76.00 (71.00,82.00)	76.00 (71.00,82.00)	76.00 (71.00,82.00)	0.891
Sex (*n*, %)				0.526
Female	4,596 (53.41)	3,203 (53.18)	1,393 (53.95)	
Male	4,009 (46.59)	2,820 (46.82)	1,189 (46.05)	
Insurance type (*n*, %)				0.046
URMI	1,318 (15.32)	937 (15.56)	381 (14.76)	
UEMI	6,506 (75.61)	4,571 (75.89)	1935 (74.94)	
Full self-pay	551 (6.40)	369 (6.13)	182 (7.05)	
Other insurance	230 (2.67)	146 (2.42)	84 (3.25)	
Marriage (*n*, %)				0.752
Married	7,858 (91.32)	5,494 (91.22)	2,364 (91.56)	
Unmarried	72 (0.84)	53 (0.88)	19 (0.74)	
DSW	675 (7.84)	476 (7.90)	199 (7.70)	
PSH (*n*, %)				0.515
Yes	5,406 (62.82)	3,770 (62.59)	1,636 (63.36)	
No	3,199 (37.18)	2,253 (37.41)	946 (36.64)	
PMH (*n*, %)				0.538
Yes	8,376 (97.34)	5,858 (97.26)	2,518 (97.52)	
No	229 (2.66)	165 (2.74)	64 (2.48)	
Smoking history (*n*, %)				0.658
Yes	2,620 (30.45)	1843 (30.60)	777 (30.09)	
No	5,985 (69.55)	4,180 (69.40)	1805 (69.91)	
Drinking history (*n*, %)				0.156
Yes	2,111 (24.53)	1,504 (24.97)	607 (23.51)	
No	6,494 (75.47)	4,519 (75.03)	1975 (76.49)	
ACCI score				0.994
≤8	5,931 (68.93)	4,152 (68.94)	1779 (68.90)	
>8	2,674 (31.07)	1871 (31.06)	803 (31.10)	
Hypertension (*n*, %)				0.095
Yes	7,022 (81.60)	4,887 (81.14)	2,135 (82.69)	
No	1,583 (18.40)	1,136 (18.86)	447 (17.31)	
CHD (*n*, %)				0.955
Yes	3,627 (42.15)	2,537 (42.12)	1,090 (42.22)	
No	4,978 (57.85)	3,486 (57.88)	1,492 (57.78)	
OP (*n*, %)				1.000
Yes	1882 (21.87)	1,317 (21.87)	565 (21.88)	
No	6,723 (78.13)	4,706 (78.13)	2017 (78.12)	
Hyperlipidemia (*n*, %)				0.556
Yes	1,427 (16.58)	989 (16.42)	438 (16.96)	
No	7,178 (83.42)	5,034 (83.58)	2,144 (83.04)	
HF (*n*, %)				0.779
Yes	2,716 (31.56)	1895 (31.46)	821 (31.80)	
No	5,889 (68.44)	4,128 (68.54)	1761 (68.20)	
PI (*n*, %)				0.403
Yes	874 (10.16)	623 (10.34)	251 (9.72)	
No	7,731 (89.84)	5,400 (89.66)	2,331 (90.28)	
Analgesics use (*n*, %)				1.000
Yes	2008 (23.34)	1,405 (23.33)	603 (23.35)	
No	6,597 (76.66)	4,618 (76.67)	1979 (76.65)	
Antibiotics use (*n*, %)				0.798
Yes	3,117 (36.22)	2,176 (36.13)	941 (36.44)	
No	5,488 (63.78)	3,847 (63.87)	1,641 (63.56)	
Psychotropic drug use (*n*, %)				0.567
Yes	3,110 (36.14)	2,189 (36.34)	921 (35.67)	
No	5,495 (63.86)	3,834 (63.66)	1,661 (64.33)	
SBP (IQR, mmHg)	140.00 (127.00,155.00)	140.00 (127.00,155.00)	140.00 (128.00,156.00)	0.378
DBP (IQR, mmHg)	78.00 (70.00,85.00)	78.00 (70.00,85.00)	78.00 (70.00,85.00)	0.948
TBIL (IQR, umol/l)	10.00 (7.60,13.30)	10.00 (7.60,13.30)	10.10 (7.61,13.40)	0.332
AKP (IQR, IU/l)	75.00 (61.59,92.00)	75.00 (61.00,92.00)	74.36 (62.00,91.00)	0.706
DBIL (IQR, umol/l)	3.75 (2.70,5.08)	3.71 (2.70,5.08)	3.80 (2.70,5.09)	0.897
AST (IQR, IU/l)	19.05 (16.00,25.00)	19.40 (16.00,25.00)	19.00 (15.64,24.00)	0.011
ALT (IQR, IU/l)	17.00 (12.00,24.00)	17.00 (12.00,24.00)	17.00 (12.00,24.00)	0.361
TC (IQR, mmol/l)	4.12 (3.39,4.95)	4.14 (3.41,4.95)	4.10 (3.35,4.97)	0.751
TGs (IQR, mmol/l)	1.36 (0.99,1.91)	1.36 (0.99,1.90)	1.36 (0.98,1.94)	0.824
NLR	3.23 (2.20,5.22)	3.23 (2.20,5.27)	3.22 (2.16,5.12)	0.359
PLR	127.42 (93.19,177.36)	128.94 (93.67,179.11)	123.96 (92.11,174.39)	0.024
LMR	3.68 (2.44,5.25)	3.68 (2.43,5.24)	3.67 (2.45,5.29)	0.638
CREA (IQR, umol/l)	74.70 (59.90,98.90)	74.95 (59.60,99.50)	74.20 (60.60,97.30)	0.586
UA (IQR, umol/l)	330.30 (265.30,410.20)	331.90 (266.30,411.20)	327.35 (263.30,407.15)	0.14
LDL-C (IQR, mmol/l)	2.24 (1.67,2.91)	2.24 (1.69,2.90)	2.24 (1.65,2.96)	0.971
HDL-C (IQR, mmol/l)	1.10 (0.92,1.32)	1.10 (0.93,1.33)	1.09 (0.91,1.32)	0.057
ALB (IQR, g/l)	39.33 (36.40,42.30)	39.30 (36.50,42.30)	39.40 (36.23,42.20)	0.292
FBG (IQR, mmol/l)	7.73 (6.01,10.77)	7.72 (6.03,10.86)	7.75 (6.00,10.64)	0.564

LOS, length of stay; URMI, urban resident medical insurance; UEMI, urban employee medical insurance; DSW, divorced & separated & widowed; PSH, past surgical history; PMH, past medical history; ACCI, age-adjusted Charlson comorbidity index; CHD, coronary heart disease; OP, osteoporosis; HF, heart failure; PI, pulmonary infection; SBP, systolic blood pressure; DBP, diastolic blood pressure; TBIL, total bilirubin; AKP, alkaline phosphatase; DBIL, direct bilirubin; AST, aspartate aminotransferase; ALT, alanine aminotransferase; TC, total cholesterol; TGs, triglycerides; NLR, neutrophil-lymphocyte ratio; PLR, platelet-lymphocyte ratio; LMR, lymphocyte-monocyte ratio; CREA, creatinine; UA, uric acid; LDL-C, low density lipoprotein cholesterol; HDL-C, high density lipoprotein cholesterol; ALB, albumin; FBG, fasting blood glucose; IQR, interquartile range.

### 3.2 Selection of predictors

Patients in the training set were divided into the normal LOS and prolonged LOS groups. The univariate analysis revealed that the following factors were significantly associated with prolonged LOS: age, sex, insurance type, ACCI score, hypertension, OP, PI, analgesics use, antibiotics use, psychotropic drug use, AKP, DBIL, TC, TGs, NLR, LMR, PLR, CREA, LDL-C, HDL-C, and ALB (*p* < 0.05) (Table 2).

**Table 2:** Demographic and clinical characteristics associated with prolonged LOS as assessed in the training set.

Variables	Total (*N* = 6,023)	Normal LOS (*N* = 1,567)	Prolonged LOS (*N* = 4,456)	*p* values
Age	76.00 (71.00,82.00)	77.00 (71.00,82.50)	76.00 (70.00,81.00)	<0.001
Sex (*n*, %)				0.028
Female	3,203 (53.18)	796 (50.80)	2,407 (54.02)	
Male	2,820 (46.82)	771 (49.20)	2049 (45.98)	
Insurance type (*n*, %)				<0.001
URMI	937 (15.56)	156 (9.96)	781 (17.53)	
UEMI	4,571 (75.89)	1,272 (81.17)	3,299 (74.04)	
Full self-pay	369 (6.13)	99 (6.32)	270 (6.05)	
Other insurance	146 (2.42)	40 (2.55)	106 (2.38)	
Marriage (*n*, %)				0.690
Married	5,494 (91.22)	1,432 (91.38)	4,062 (91.16)	
Unmarried	53 (0.88)	16 (1.02)	37 (0.83)	
DSW	476 (7.90)	119 (7.60)	357 (8.01)	
PSH (*n*, %)				0.307
Yes	3,770 (62.59)	964 (61.52)	2,806 (62.97)	
No	2,253 (37.41)	603 (38.48)	1,650 (37.03)	
PMH (*n*, %)				0.146
Yes	5,858 (97.26)	1,516 (96.75)	4,342 (97.44)	
No	165 (2.74)	51 (3.25)	114 (2.56)	
Smoking history (*n*, %)				0.191
Yes	1843 (30.60)	500 (31.91)	1,343 (30.14)	
No	4,180 (69.40)	1,067 (68.09)	3,113 (69.86)	
Drinking history (*n*, %)				0.510
Yes	1,504 (24.97)	401 (25.59)	1,103 (24.75)	
No	4,519 (75.03)	1,166 (74.41)	3,353 (75.25)	
ACCI score				<0.001
≤8	4,152 (68.94)	981 (62.60)	3,171 (71.16)	
>8	1871 (31.06)	586 (37.40)	1,285 (28.84)	
Hypertension (*n*, %)				<0.001
Yes	4,887 (81.14)	1,324 (84.49)	3,563 (79.96)	
No	1,136 (18.86)	243 (15.51)	893 (20.04)	
CHD (*n*, %)				0.066
Yes	2,537 (42.12)	691 (44.10)	1846 (41.43)	
No	3,486 (57.88)	876 (55.90)	2,610 (58.57)	
OP (*n*, %)				<0.001
Yes	1,317 (21.87)	438 (27.95)	879 (19.73)	
No	4,706 (78.13)	1,129 (72.05)	3,577 (80.27)	
Hyperlipidemia (*n*, %)				0.329
Yes	989 (16.42)	245 (15.63)	744 (16.70)	
No	5,034 (83.58)	1,322 (84.37)	3,712 (83.30)	
HF (*n*, %)				0.185
Yes	1895 (31.46)	514 (32.80)	1,381 (30.99)	
No	4,128 (68.54)	1,053 (67.20)	3,075 (69.01)	
PI (*n*, %)				<0.001
Yes	623 (10.34)	263 (16.78)	360 (8.08)	
No	5,400 (89.66)	1,304 (83.22)	4,096 (91.92)	
Analgesics use (*n*, %)				<0.001
Yes	1,405 (23.33)	598 (38.16)	807 (18.11)	
No	4,618 (76.67)	969 (61.84)	3,649 (81.89)	
Antibiotics use (*n*, %)				<0.001
Yes	2,176 (36.13)	830 (52.97)	1,346 (30.21)	
No	3,847 (63.87)	737 (47.03)	3,110 (69.79)	
Psychotropic drug use (*n*, %)				<0.001
Yes	2,189 (36.34)	823 (52.52)	1,366 (30.66)	
No	3,834 (63.66)	744 (47.48)	3,090 (69.34)	
SBP (IQR, mmHg)	140.00 (127.00,155.00)	138.00 (126.00,155.00)	140.00 (128.00,155.00)	0.086
DBP (IQR, mmHg)	78.00 (70.00,85.00)	77.00 (70.00,85.00)	78.00 (70.00,85.00)	0.309
TBIL (IQR, umol/l)	10.00 (7.60,13.30)	10.00 (7.55,13.42)	10.00 (7.60,13.20)	0.735
AKP (IQR, IU/l)	75.00 (61.00,92.00)	76.00 (61.93,94.66)	74.00 (61.00,91.23)	0.015
DBIL (IQR, umol/l)	3.71 (2.70,5.08)	4.00 (2.90,5.40)	3.70 (2.70,4.96)	<0.001
AST (IQR, IU/l)	19.40 (16.00,25.00)	19.00 (15.52,25.00)	19.70 (16.00,25.00)	0.156
ALT (IQR, IU/l)	17.00 (12.00,24.00)	17.00 (11.70,25.00)	17.00 (12.00,24.00)	0.932
TC (IQR, mmol/l)	4.14 (3.41,4.95)	4.06 (3.32,4.84)	4.17 (3.44,4.98)	<0.001
TGs (IQR, mmol/l)	1.36 (0.99,1.90)	1.29 (0.93,1.79)	1.39 (1.01,1.93)	<0.001
NLR	3.23 (2.20,5.27)	3.54 (2.36,6.29)	3.13 (2.17,5.01)	<0.001
PLR	128.94 (93.67,179.11)	135.29 (99.06,189.11)	126.37 (92.24,175.00)	<0.001
LMR	3.68 (2.43,5.24)	3.34 (2.22,4.80)	3.79 (2.54,5.41)	<0.001
CREA (IQR, umol/l)	74.95 (59.60,99.50)	77.50 (59.91,106.40)	74.40 (59.48,97.00)	0.001
UA (IQR, umol/l)	331.90 (266.30,411.20)	330.70 (257.40,413.95)	332.10 (269.80,410.23)	0.297
LDL-C (IQR, mmol/l)	2.24 (1.69,2.90)	2.19 (1.62,2.84)	2.26 (1.71,2.93)	0.001
HDL-C (IQR, mmol/l)	1.10 (0.93,1.33)	1.08 (0.90,1.32)	1.11 (0.94,1.33)	0.006
ALB (IQR, g/l)	39.30 (36.50,42.30)	38.49 (35.30,41.60)	39.60 (36.90,42.50)	<0.001
FBG (IQR, mmol/l)	7.72 (6.03,10.86)	7.77 (6.01,11.02)	7.68 (6.03,10.77)	0.583

URMI, urban resident medical insurance; UEMI, urban employee medical insurance; DSW, divorced & separated & widowed; PSH, past surgical history; PMH, past medical history; ACCI, age-adjusted Charlson comorbidity index; CHD, coronary heart disease; OP, osteoporosis; HF, heart failure; PI, pulmonary infection; SBP, systolic blood pressure; DBP, diastolic blood pressure; TBIL, total bilirubin; AKP, alkaline phosphatase; DBIL, direct bilirubin; AST, aspartate aminotransferase; ALT, alanine aminotransferase; TC, total cholesterol; TGs, triglycerides; NLR, neutrophil-lymphocyte ratio; PLR, platelet-lymphocyte ratio; LMR, lymphocyte-monocyte ratio; CREA, creatinine; UA, uric acid; LDL-C, low density lipoprotein cholesterol; HDL-C, high density lipoprotein cholesterol; ALB, albumin; FBG, fasting blood glucose; LOS, length of stay; IQR, interquartile range.

**Figure 1:**
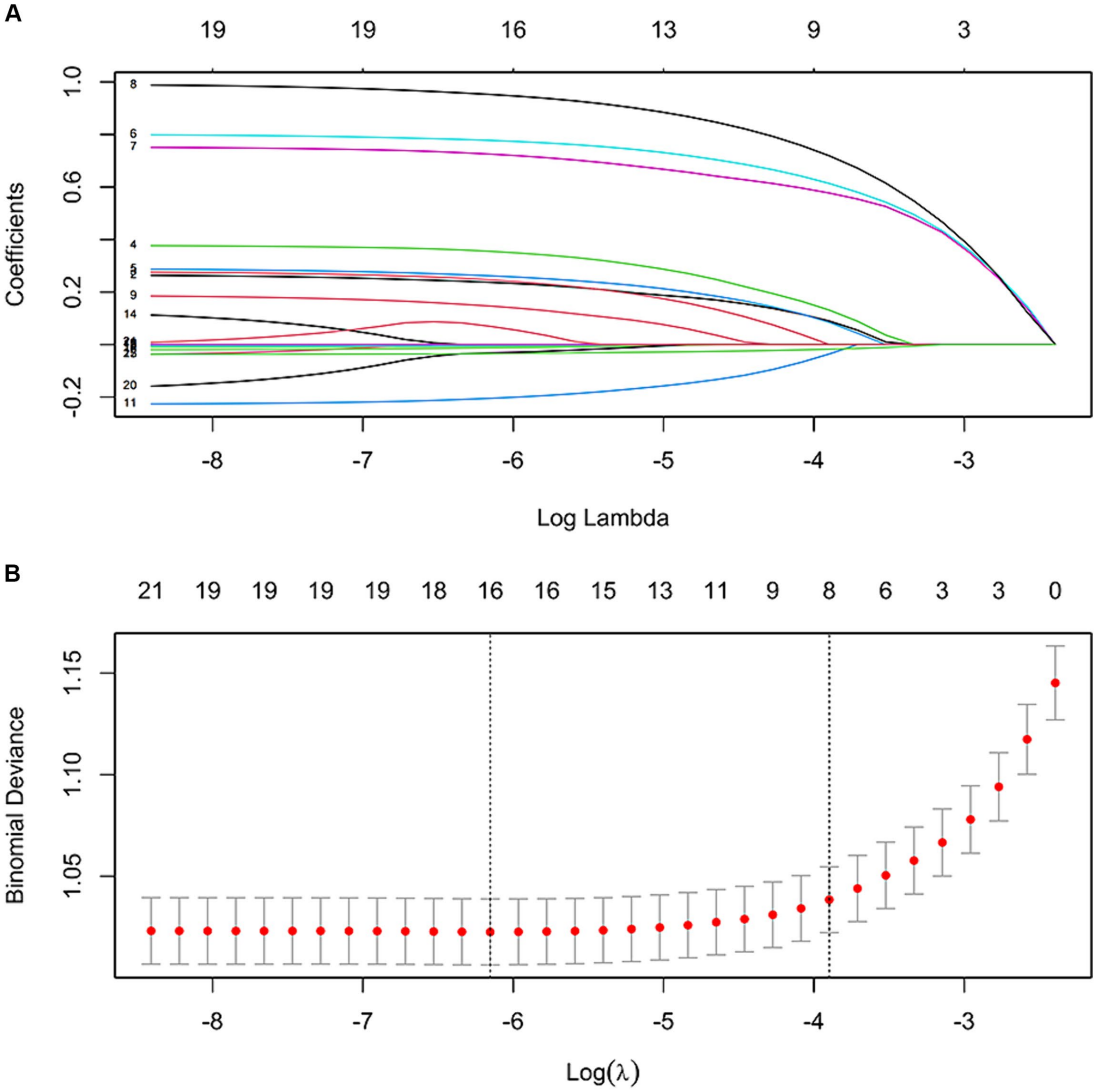
Features selection by LASSO. **(A)** LASSO coefficient profile (*y*-axis) of the 21 features. The upper *x*-axis represents the average number of predictors and the lower *x*-axis is the log (*λ*). **(B)** Tenfold cross-validation for tuning parameter selection in the LASSO model.

**Figure 2:**
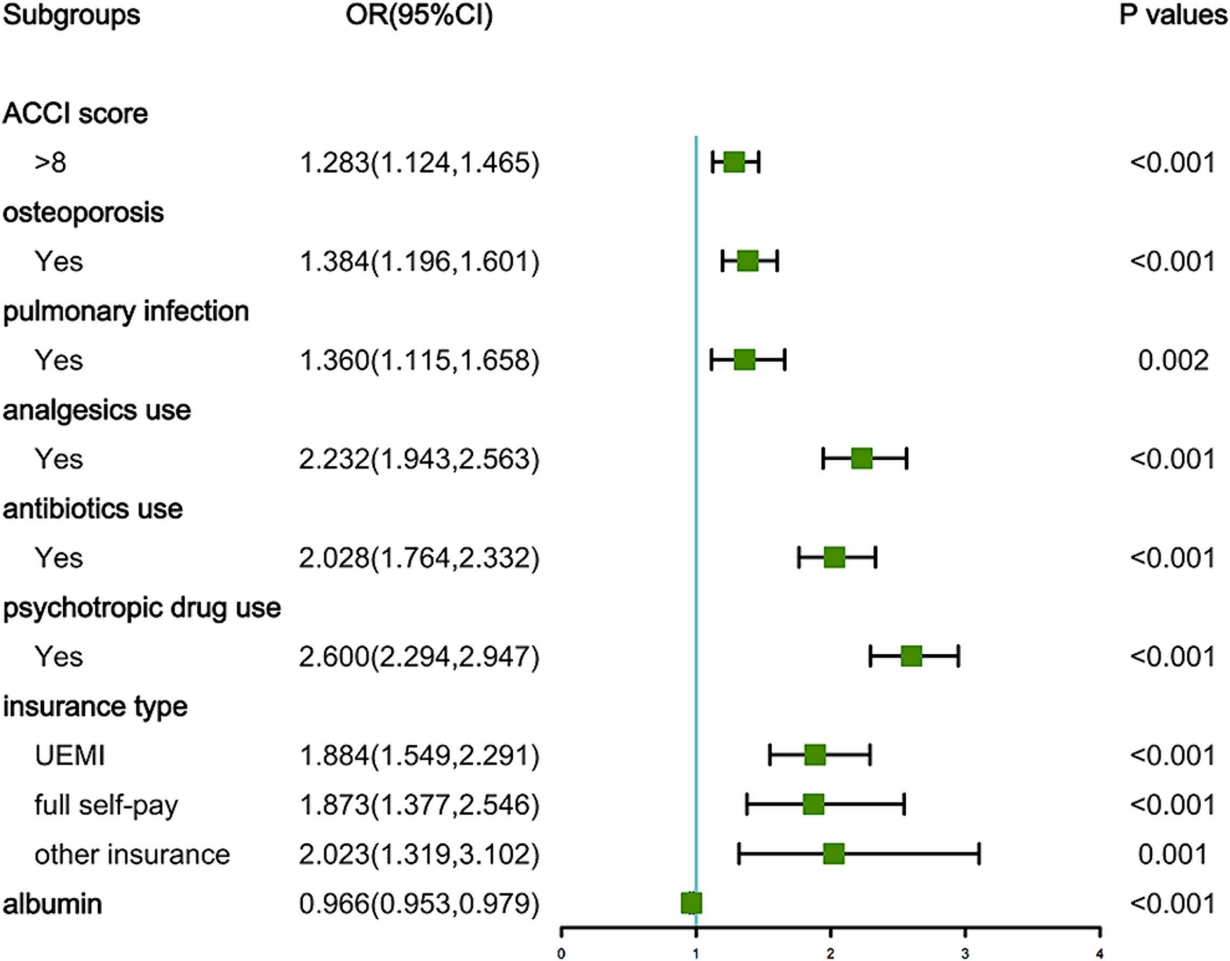
Forest plot showing the results of multivariable analysis.

As depicted in Figure 1, the LASSO regression utilized a Lambda value of 0.02028737 and yielded eight predictors, namely the ACCI score, OP, PI, analgesics use, antibiotics use, psychotropic drug use, insurance type, and ALB. Ultimately, the multivariate logistic regression model revealed that the ACCI score (odds ratio [OR]: 1.283, 95% confidence interval [CI]: 1.124–1.465, *p* < 0.001), OP (OR: 1.384, 95% CI: 1.196–1.601, *p* < 0.001), PI (OR: 1.360, 95% CI: 1.115–1.658, *p* = 0.002), analgesics use (OR: 2.232, 95% CI: 1.943–2.563, *p* < 0.001), antibiotics use (OR: 2.028, 95% CI: 1.764–2.332, *p* < 0.001), psychotropic drug use (OR: 2.600, 95% CI: 2.294–2.947, *p* < 0.001), insurance type ([UEMI], OR: 1.884, 95% CI: 1.549–2.291, *p* < 0.001; [full self-pay], OR: 1.873, 95% CI: 1.377–2.546, *p* < 0.001; [other insurance], OR: 2.023, 95% CI: 1.319–3.102, *p* = 0.001), and ALB (OR: 0.966, 95% CI: 0.953–0.979, *p* < 0.001) were independent predictors of prolonged LOS risk (Figure 2).

### 3.3 Nomogram construction and performance

**Figure 3:**
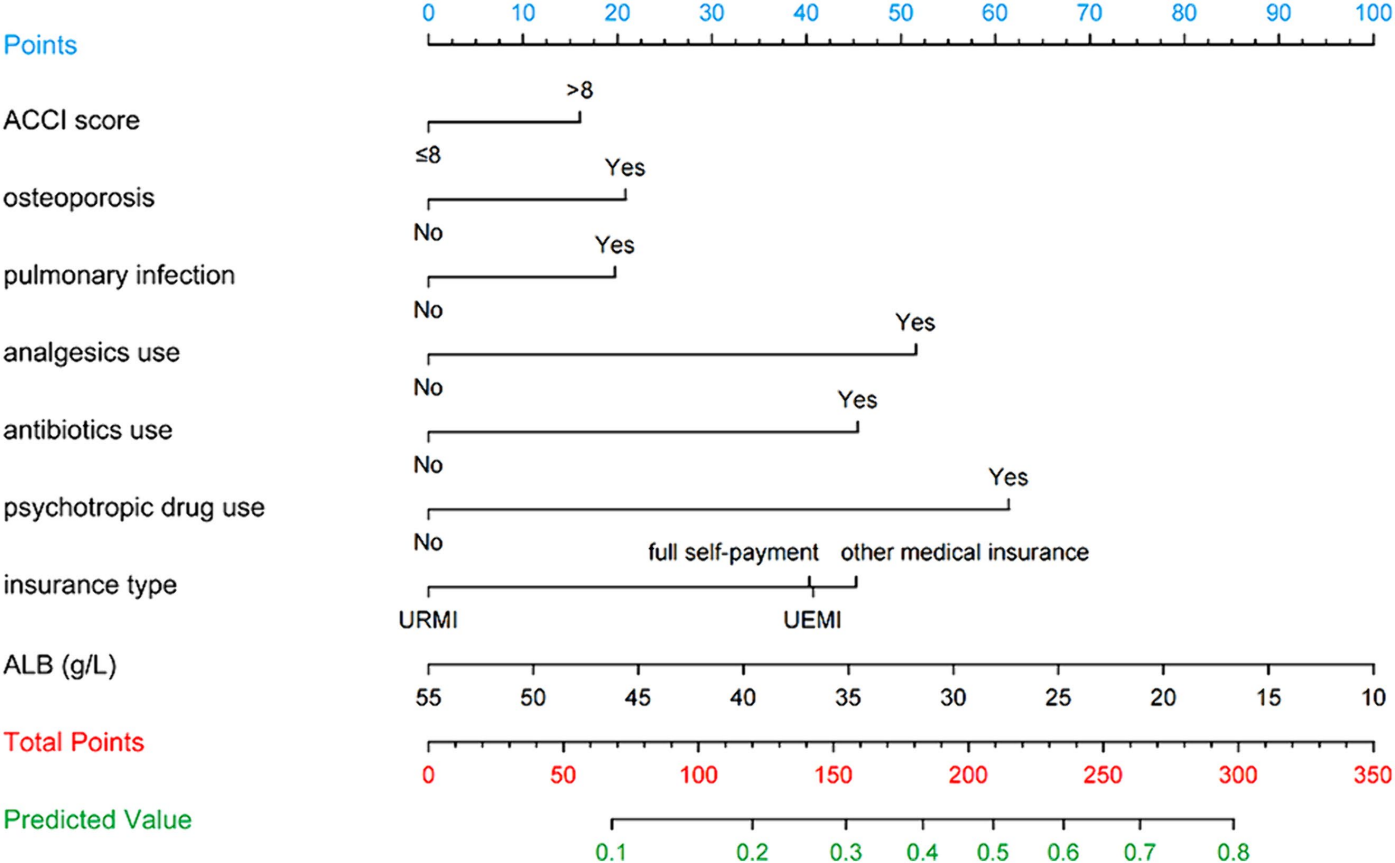
Nomogram predicting prolonged LOS in elderly T2DM patients complicated with CI.

**Figure 4:**
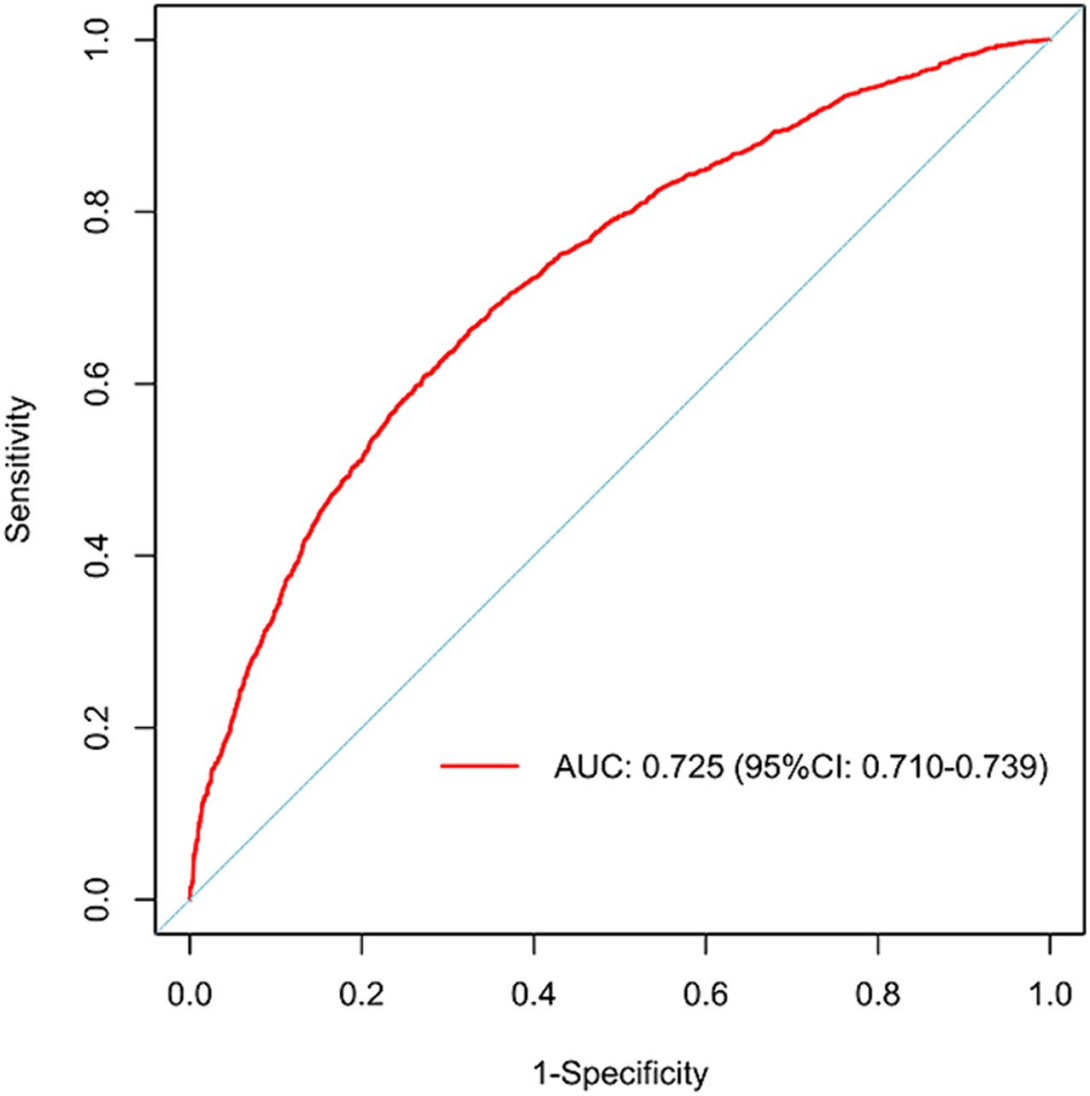
AUROC of the model in the training set.

**Figure 5:**
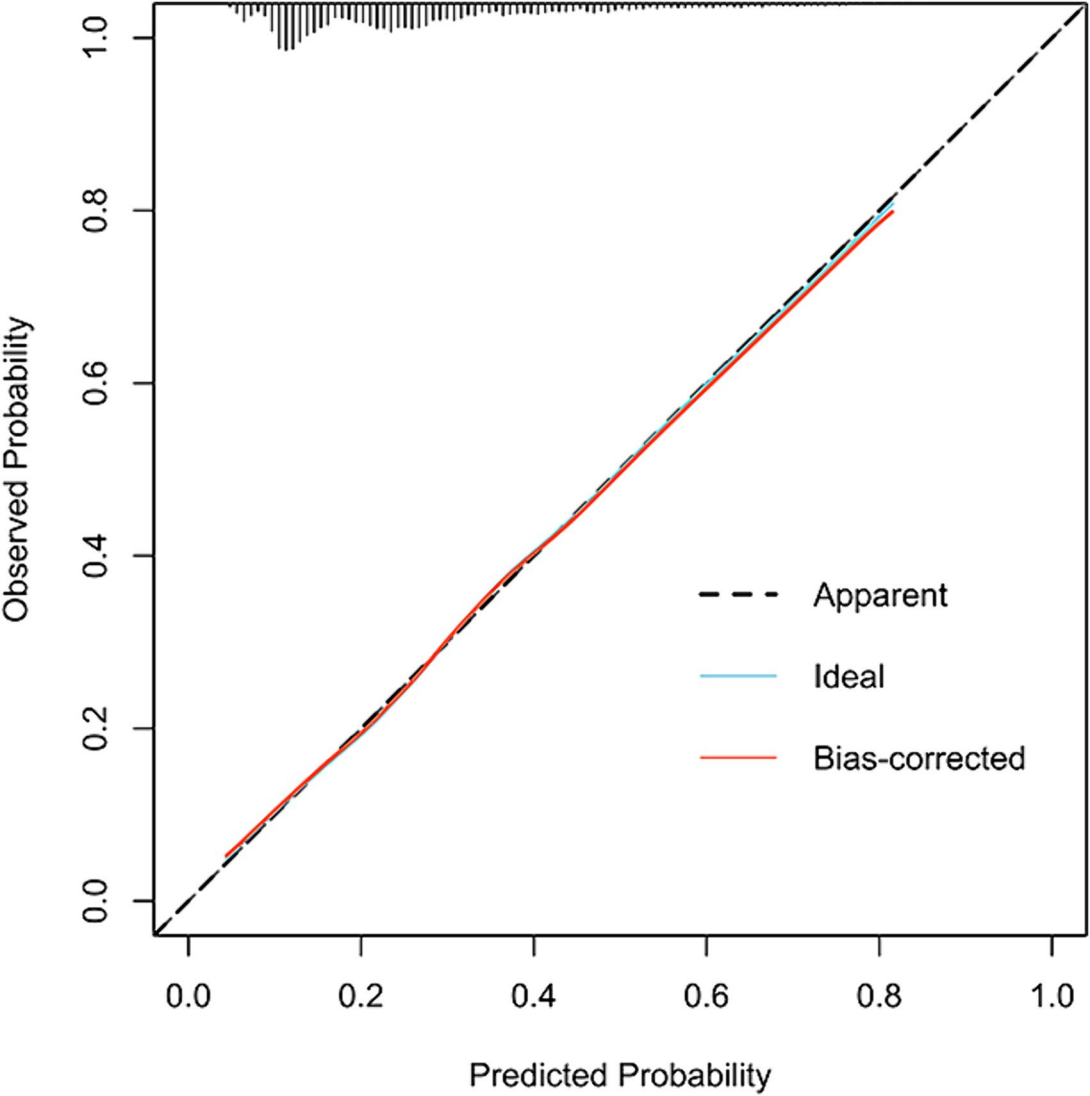
The calibration curve of the model in the training set.

Figure 3 illustrates the LASSO logistic regression model as a nomogram for calculating the probability of prolonged LOS in elderly T2DM patients complicated with CI. The model displayed a high predictive ability, with an AUROC of 0.725 (95% CI: 0.710–0.739) in the training set (Figure 4), 0.707 (95% CI: 0.684–0.730) in the internal validation set, and 0.691 (95% CI: 0.670–0.712) in the external validation set. The best cutoff value was 0.256. Moreover, the calibration curve of the training set indicated that the predicted probability was consistent with the observed probability, demonstrating the reliable predictive ability of the nomogram (Figure 5). Supplementary Figures S2, S3 display the AUROCs for the internal and external validation sets, respectively. Supplementary Figures S4, S5 illustrate the calibration curves for the internal and external validation sets, respectively. Table 3 presents the detailed performance metrics for the three sets.

**Table 3:** Detailed performance metrics of the three sets.

Models	AUROC	Sensitivity	Specificity
	(95%CI)	(95%CI)	(95%CI)
Training set	0.725	0.662	0.675
	0.710–0.739	0.639–0.686	0.661–0.689
Internal validation set	0.707	0.627	0.688
	0.684–0.730	0.590–0.665	0.667–0.708
External validation set	0.691	0.673	0.619
	0.670–0.712	0.640–0.706	0.604–0.634

AUROC, area under the receiver operating characteristic curve; CI, confidence interval.

### 3.4 Clinical utility of the nomogram

**Figure 6:**
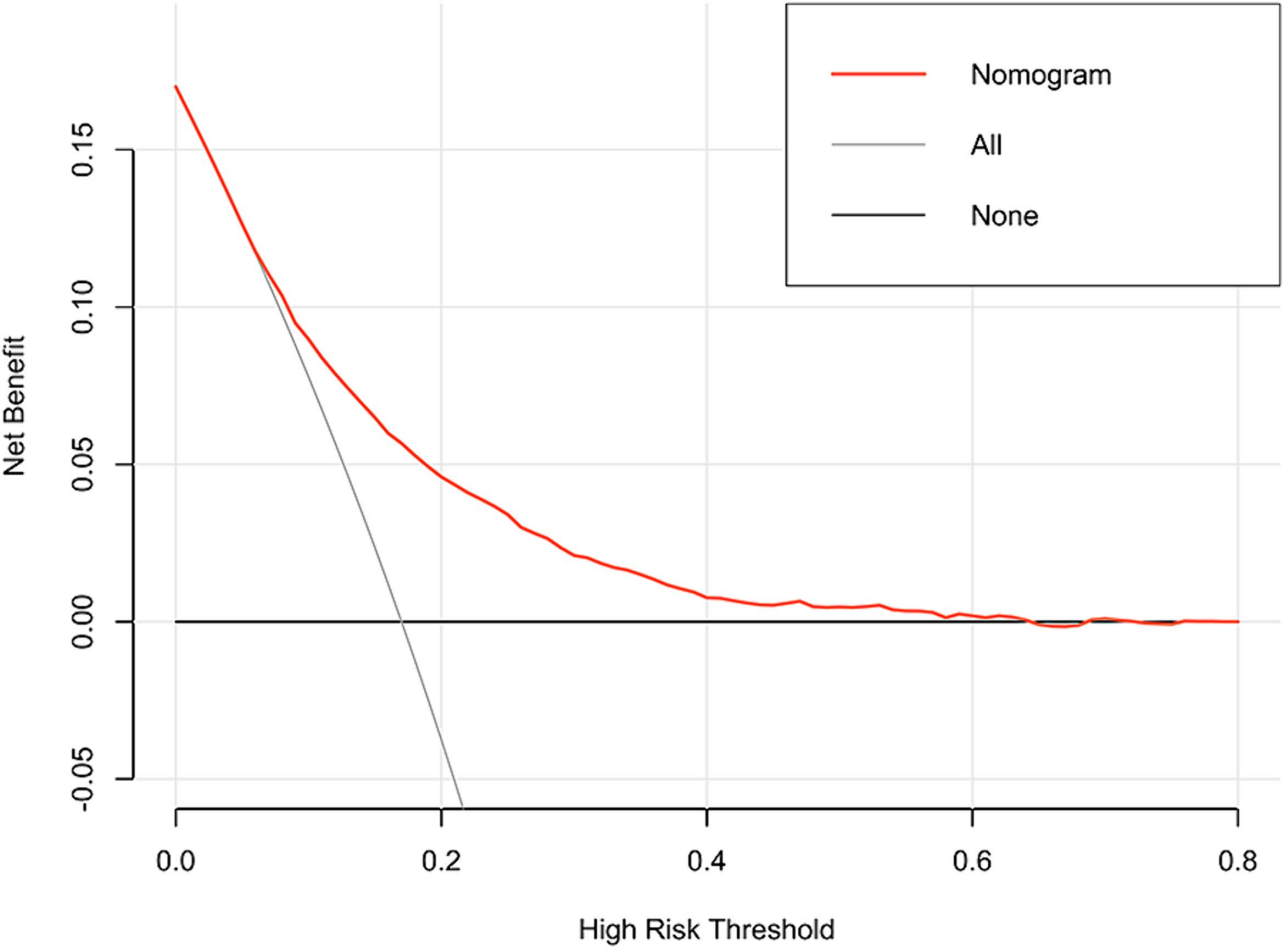
DCA of the model in the training set.

**Figure 7:**
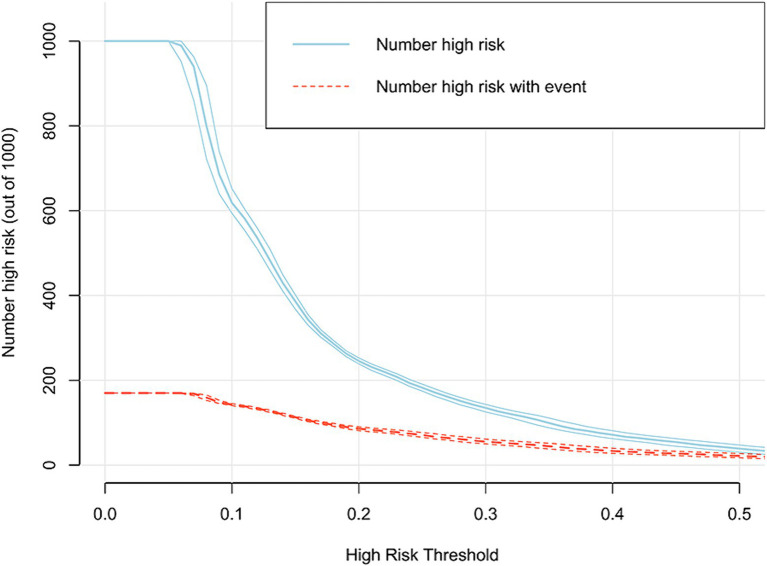
CIC of the model in the training set.

In addition, the DCA reflected favorable potential clinical effects. When the prediction probability threshold was set at 8–63%, the net benefit range was 0–12% (Figure 6). Meanwhile, the CIC indicated that the model yielded superior overall net returns within the threshold probability range (Figure 7). Supplementary Figures S6, S7 illustrate the DCAs for the internal and external validation sets, respectively. Supplementary Figures S8, S9 display the CICs for the internal and external validation sets, respectively.

### 3.5 Construction of the web app to access the nomogram

**Figure 8:**
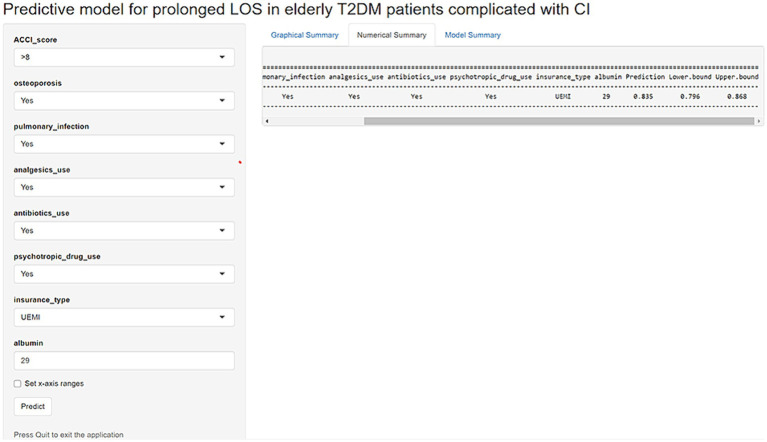
An example of a model predicting prolonged LOS in elderly T2DM patients complicated with CI via a link.

To facilitate the clinical use of the model, an operation interface was constructed on a web page^1^ to calculate the exact probability of prolonged LOS. For example, for a patient having OP, PI, analgesics use, antibiotics use, psychotropic drug use, ACCI score > 8, insurance type of UEMI, and ALB level of 29.00 g/L, the probability of prolonged LOS would be 0.835 (95% CI: 0.796–0.868) (Figure 8).

## 4 Discussion

LOS is an important indicator that directly reflects the quality of hospital services and the utilization of medical resources (19). Prolonged LOS may have adverse effects on the economic and social benefits of hospitals (20). Moreover, shortening the LOS reduces the economic burden and improves patients’ quality of life, while accelerating the turnover speed of hospital beds, thereby enhancing the social and economic benefits of the hospital. This study investigated a large number of characteristics and clinical data that may be associated with an increased risk of prolonged LOS in elderly T2DM patients complicated with CI. An easy-to-use predictive model was developed based on eight predictors (ACCI score, OP, PI, analgesics use, antibiotics use, psychotropic drug use, insurance type, and ALB) to identify the risk of prolonged LOS. The model exhibited an AUROC of 0.725 (95% CI: 0.710–0.739), a sensitivity of 0.662 (95% CI: 0.639–0.686), and a specificity of 0.675 (95% CI: 0.661–0.689).

The new risk model showed a significant advantage compared to the previously developed models for predicting the risk of prolonged LOS in elderly patients (21–23). Many of the data elements used for risk adjustment were identical, including infections, drug use, and ALB. However, the main advantage of the new model was the ability to measure multiple comorbidities after admission.

The results of this study suggested that the ACCI score (OR: 1.283, 95% CI: 1.124–1.465) was a risk factor for prolonged LOS in elderly T2DM patients complicated with CI. The ACCI score reflects the overall health status of patients upon admission. Lanny et al. reported that the number of comorbidities was associated with an increased LOS (*r* = 0.4596, *p* < 0.001). Having at least one comorbidity was associated with a 13% greater LOS (IRR = 1.13, 95% CI: 1.11–1.15, *p* < 0.001) (24). Another study also revealed that patients with ≥3 comorbid conditions were three times more likely to die (OR: 3.03, 95% CI: 1.40–6.20, *p* < 0.01) and six times more likely to have prolonged LOS (OR: 6.40, 95%CI: 4.80–8.60, *p* < 0.001) compared to patients with ≤1 comorbid condition (25). Patients with severe comorbidities require multiple medications and are prone to complications, which can prolong the patient’s LOS (26, 27). In addition, these patients have low self-care ability and require a longer period to recover to their initial level of self-care. Based on the results of this study, medical staff should pay particular attention to patients with ACCI scores>8.

The ALB level is a common indicator used to evaluate the nutritional status in clinical practice, which reflects various physiological functions, such as maintaining a constant plasma colloid osmotic pressure, material transport, regulating hormone and drug metabolism, etc. (28–30). The normal range of ALB in adults is 35–50 g/L (31). In this study, the ALB content in most elderly T2DM patients complicated with CI was lower than the normal range, indicating that malnutrition is common in elderly T2DM patients complicated with CI. Studies have shown that abnormal ALB levels were associated with decreased respiratory muscle endurance, impaired immune function, and increased risk of CI and respiratory failure (32, 33). Therefore, attention should be paid to the ALB levels of elderly T2DM patients complicated with CI, and nutritional intervention plans should be developed to prevent prolonged LOS.

Besides, OP, PI, and insurance type have been proven to be risk factors for prolonged LOS, which was consistent with the results of this study (34–36). Interestingly, our results also revealed that analgesics use (dexamethasone, betamethasone, methylprednisolone, and others), antibiotics use (levofloxacin hydrochloride, amoxicillin capsules, metronidazole tablets, and others), and psychotropic drug use (estazolam tablets, phenobarbital tablets, quetiapine tablets, and others) were correlated with prolonged LOS in elderly T2DM patients complicated with CI. The underlying conditions associated with the use of drugs may be correlated with prolonged LOS; however, further research is needed to confirm the effects of certain drugs on prolonged LOS.

The advantages of this study mainly include the use of a large sample and multicenter data to construct the prediction model, and the use of readily available variables to construct the predictive model, which greatly improves the model’s generalizability and facilitates its application to clinical practice. Nevertheless, the limitations of the present study should be acknowledged. First, the study adopted a retrospective design, providing weaker evidence compared with prospective studies. Hence, the findings should be interpreted with caution. Second, some potential influencing factors were ignored due to the high percentage of missing data. The addition of these factors could improve the prediction efficiency of the model. Therefore, prospective studies with more detailed data and larger sample sizes are needed to verify or update the findings.

## 5 Conclusion

This study analyzed the influencing factors of prolonged LOS in elderly T2DM patients complicated with CI based on multicenter EMRs data and constructed a predictive model to assess the risk of prolonged LOS. ACCI score, OP, PI, analgesics use, antibiotics use, psychotropic drug use, insurance type, and ALB were independent predictors for prolonged LOS of elderly T2DM patients complicated with CI. The prediction model constructed based on eight predictors can provide a basis to optimize hospital management, considering the risk of prolonged LOS for elderly T2DM patients complicated with CI.

## Data availability statement

The raw data supporting the conclusions of this article will be made available by the authors, without undue reservation.

## Ethics statement

The studies involving humans were approved by the Ethics Committee of the Affiliated Banan Hospital of Chongqing Medical University. The studies were conducted in accordance with the local legislation and institutional requirements. Written informed consent for participation was not required from the participants or the participants’ legal guardians/next of kin in accordance with the national legislation and institutional requirements.

## Author contributions

MT: Writing – review & editing, Writing – original draft, Funding acquisition, Formal analysis. YZ: Writing – review & editing, Writing – original draft, Data curation. JX: Writing – original draft, Methodology. SJ: Writing – review & editing, Validation, Resources, Conceptualization. JT: Writing – original draft, Methodology, Data curation. QX: Writing – original draft, Validation, Formal analysis. CP: Writing – review & editing, Validation, Supervision, Investigation, Formal analysis. JW: Writing – review & editing, Validation, Supervision, Methodology, Investigation, Formal analysis.

## Footnotes

^1^https://xxmyyz.shinyapps.io/prolonged_los1/
