# Supplementary material for: Development and validation of a predictive model for prolonged length of stay in elderly type 2 diabetes mellitus patients combined with cerebral infarction
Source: Front Neurol. 2024 Aug 1;15:1405096. doi: 10.3389/fneur.2024.1405096 (PMC11325865; doi:10.3389/fneur.2024.1405096)
Supplement: Supplementary file 1 [file Table_1.DOCX]

**Supplementary information**

**
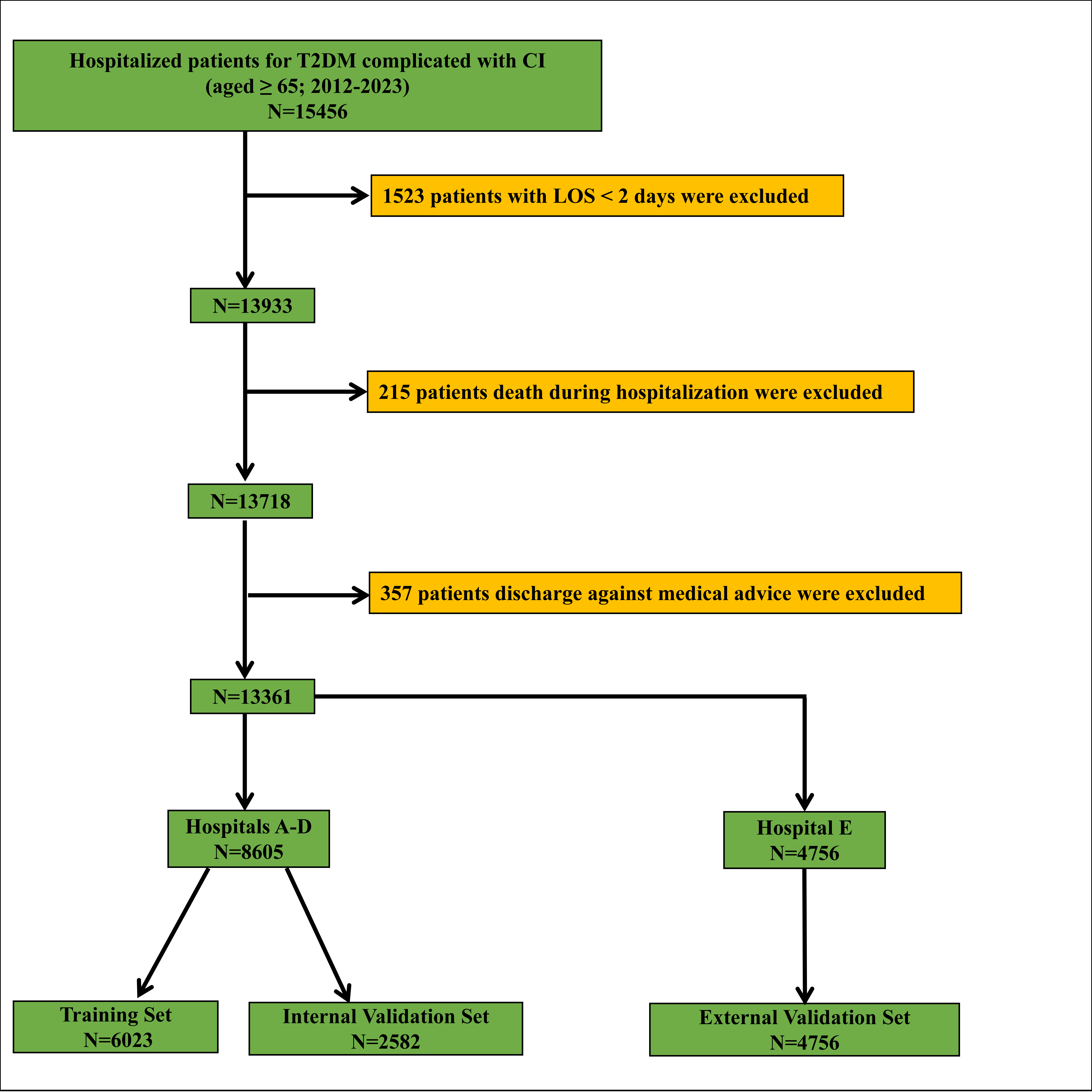
**

**Figure S1 Flow of inclusions and exclusions**

**Table S1 Information of five institutions in This Study**

| Institutions | NO.of Patients Enrolled | No. of Prolonged LOS |
| --- | --- | --- |
| Chongqing Southeast Hospital | 827 | 154 |
| Second Affiliated Hospital of Chongqing Medical University | 4763 | 1293 |
| University-Town Hospital of Chongqing Medical University | 1432 | 520 |
| Third Affiliated Hospital of Chongqing Medical University | 1583 | 255 |
| the Affiliated Banan Hospital of Chongqing Medical University | 4756 | 779 |
| Total | 13361 | 3001 |

*LOS: length of stay.*

**Table S2. Comparison of missing variables in the training set before and after multiple imputation**

| Missing variables | Before interpolation | After interpolation | *P* values |
| --- | --- | --- | --- |
| SBP (IQR, mmHg) | 140.00(127.00,155.00) | 140.00(127.00,155.00) | 0.805 |
| DBP (IQR, mmHg) | 77.00(70.00,85.00) | 78.00(70.00,85.00) | 0.694 |
| TBIL (IQR, umol/l) | 10.00(7.60,13.30) | 10.00(7.60,13.30) | 0.843 |
| AKP (IQR, IU/l) | 75.00(61.20,92.00) | 75.00(61.00,92.00) | 0.664 |
| DBIL (IQR, umol/l) | 3.73(2.70,5.08) | 3.71(2.70,5.08) | 0.988 |
| AST (IQR, IU/l) | 19.19(16.00,25.00) | 19.40(16.00,25.00) | 0.681 |
| ALT (IQR, IU/l) | 17.00(12.00,24.44) | 17.00(12.00,24.00) | 0.360 |
| TC (IQR, mmol/l) | 4.15(3.42,4.97) | 4.14(3.41,4.95) | 0.645 |
| TGs (IQR, mmol/l) | 1.38(1.00,1.91) | 1.36(0.99,1.90) | 0.309 |
| NLR | 3.25(2.21,5.30) | 3.23(2.20,5.27) | 0.697 |
| PLR | 128.89(93.68,180.00) | 128.94(93.67,179.11) | 0.913 |
| LMR | 3.67(2.42,5.22) | 3.68(2.43,5.24) | 0.827 |
| CREA (IQR, umol/l) | 75.10(59.80,99.60) | 74.95(59.60,99.50) | 0.778 |
| UA (IQR, umol/l) | 332.10(266.30,411.70) | 331.90(266.30,411.20) | 0.846 |
| LDL-C (IQR, mmol/l) | 2.25(1.69,2.92) | 2.24(1.69,2.90) | 0.605 |
| HDL-C (IQR, mmol/l) | 1.10(0.93,1.32) | 1.10(0.93,1.33) | 0.766 |
| ALB (IQR, g/l) | 39.30(36.50,42.30) | 39.30(36.50,42.30) | 0.821 |
| FBG (IQR, mmol/l) | 7.68(6.02,10.77) | 7.72(6.03,10.86) | 0.653 |

*SBP: systolic blood pressure; DBP: diastolic blood pressure; TBIL: total bilirubin; AKP: alkaline phosphatase; DBIL: direct bilirubin; AST: aspartate aminotransferase; ALT: alanine aminotransferase; TC:total cholesterol; TGs: triglycerides; NLR: neutrophil-lymphocyte ratio; PLR: platelet-lymphocyte ratio; LMR: lymphocyte-monocyte ratio; CREA: creatinine; UA:uric acid; LDL-C: low density lipoprotein cholesterol; HDL-C: high density lipoprotein cholesterol; ALB: albumin; FBG: fasting blood glucose; LOS: length of stay; IQR: interquartile range.*

**Table S3. Comparison of missing variables in the internal validation set before and after multiple imputation**

| Missing variables | Before interpolation | After interpolation | *P* values |
| --- | --- | --- | --- |
| SBP (IQR, mmHg) | 140.00(128.00,156.00) | 140.00(128.00,156.00) | 0.913 |
| DBP (IQR, mmHg) | 78.00(70.00,85.00) | 78.00(70.00,85.00) | 0.881 |
| TBIL (IQR, umol/l) | 10.10(7.60,13.40) | 10.10(7.61,13.40) | 0.945 |
| AKP (IQR, IU/l) | 74.00(62.00,91.00) | 74.36(62.00,91.00) | 0.987 |
| DBIL (IQR, umol/l) | 3.80(2.70,5.10) | 3.80(2.70,5.09) | 0.968 |
| AST (IQR, IU/l) | 19.00(15.66,24.58) | 19.00(15.64,24.00) | 0.641 |
| ALT (IQR, IU/l) | 17.00(12.00,24.00) | 17.00(12.00,24.00) | 0.852 |
| TC (IQR, mmol/l) | 4.11(3.37,4.99) | 4.10(3.35,4.97) | 0.541 |
| TGs (IQR, mmol/l) | 1.37(1.01,1.95) | 1.36(0.98,1.94) | 0.508 |
| NLR | 3.24(2.19,5.17) | 3.22(2.16,5.12) | 0.718 |
| PLR | 124.29(92.34,174.53) | 123.96(92.11,174.39) | 0.851 |
| LMR | 3.67(2.44,5.26) | 3.67(2.45,5.29) | 0.818 |
| CREA (IQR, umol/l) | 74.40(60.60,98.16) | 74.20(60.60,97.30) | 0.765 |
| UA (IQR, umol/l) | 328.60(264.35,407.80) | 327.35(263.30,407.15) | 0.671 |
| LDL-C (IQR, mmol/l) | 2.24(1.66,2.97) | 2.24(1.65,2.96) | 0.656 |
| HDL-C (IQR, mmol/l) | 1.09(0.92,1.32) | 1.09(0.91,1.32) | 0.674 |
| ALB (IQR, g/l) | 39.40(36.29,42.10) | 39.40(36.23,42.20) | 0.867 |
| FBG (IQR, mmol/l) | 7.72(5.98,10.61) | 7.75(6.00,10.64) | 0.723 |

*SBP: systolic blood pressure; DBP: diastolic blood pressure; TBIL: total bilirubin; AKP: alkaline phosphatase; DBIL: direct bilirubin; AST: aspartate aminotransferase; ALT: alanine aminotransferase; TC:total cholesterol; TGs: triglycerides; NLR: neutrophil-lymphocyte ratio; PLR: platelet-lymphocyte ratio; LMR: lymphocyte-monocyte ratio; CREA: creatinine; UA:uric acid; LDL-C: low density lipoprotein cholesterol; HDL-C: high density lipoprotein cholesterol; ALB: albumin; FBG: fasting blood glucose; LOS: length of stay; IQR: interquartile range.*

**
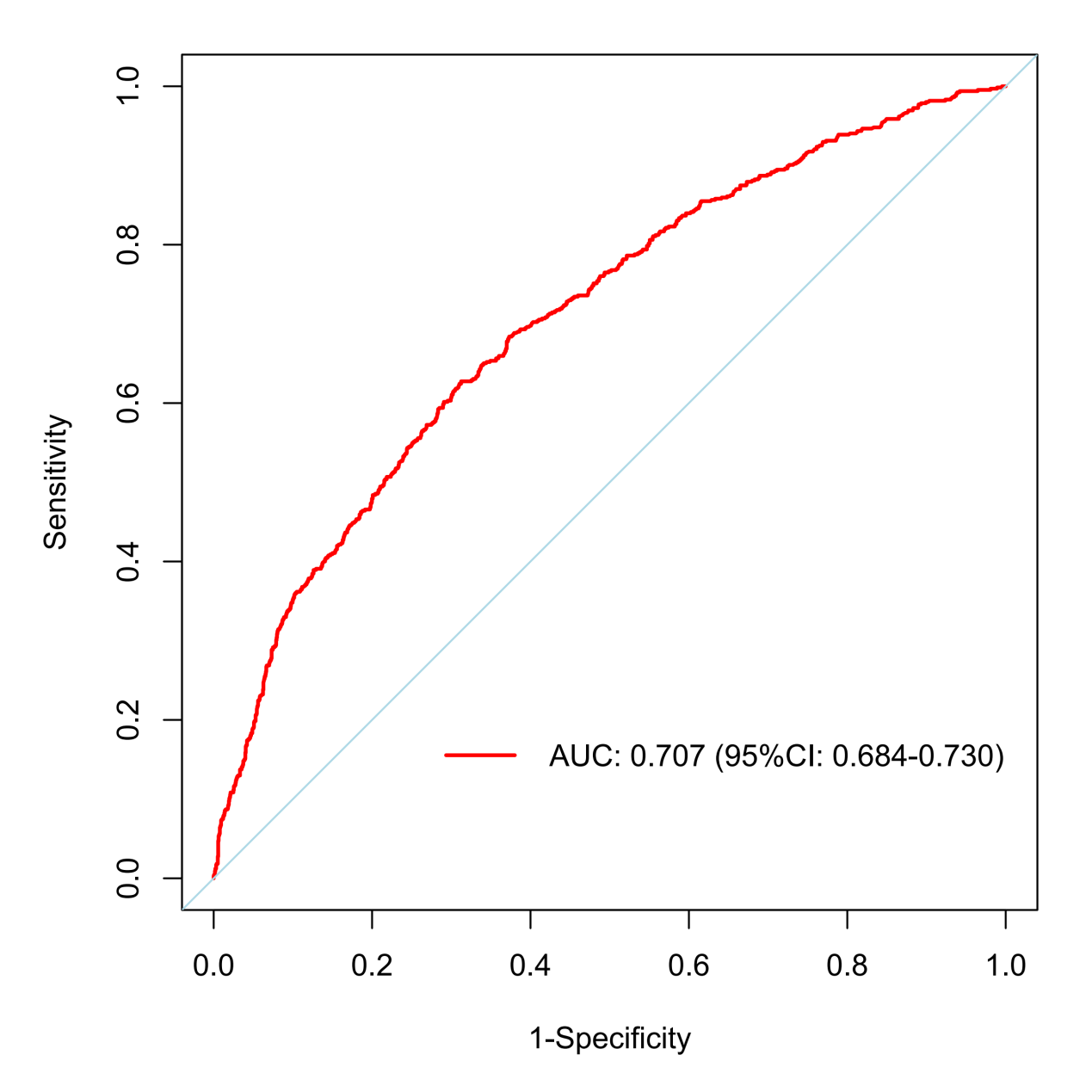
**

**Figure S2 AUROC of the model in the internal validation set**

**
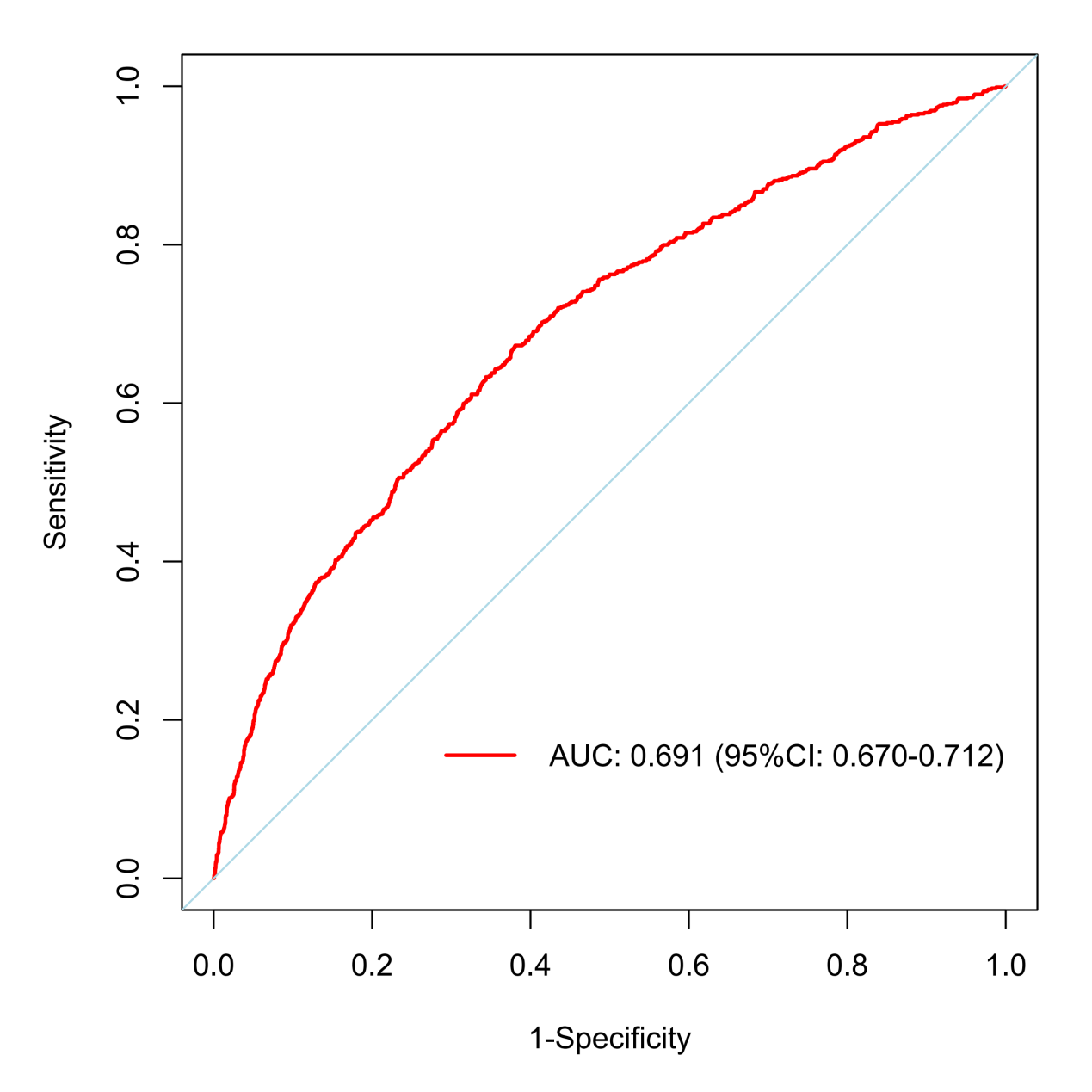
**

**Figure S3 AUROC of the model in the external validation set**

**
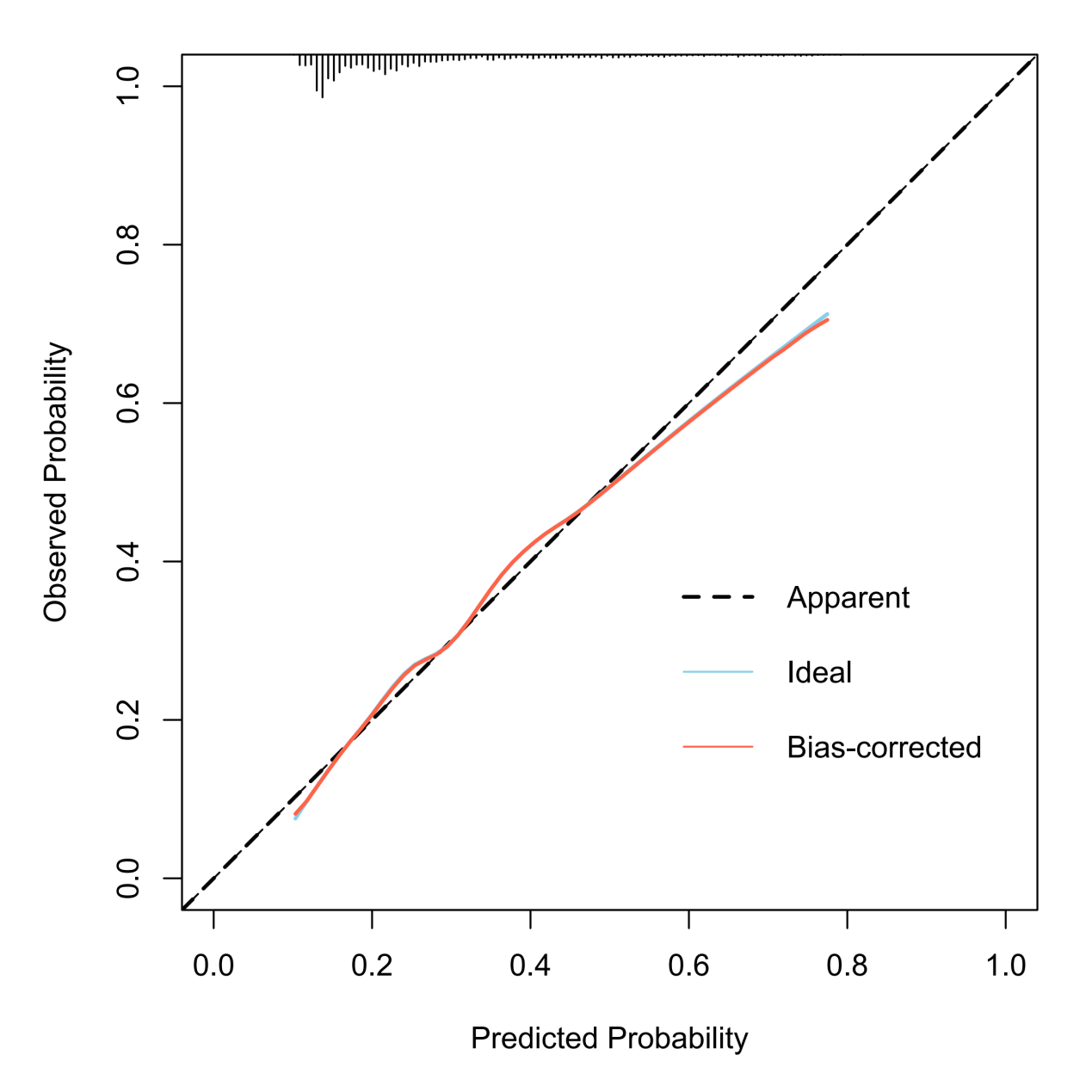
**

**Figure S4 Calibration curve of the model in the internal validation set**

**
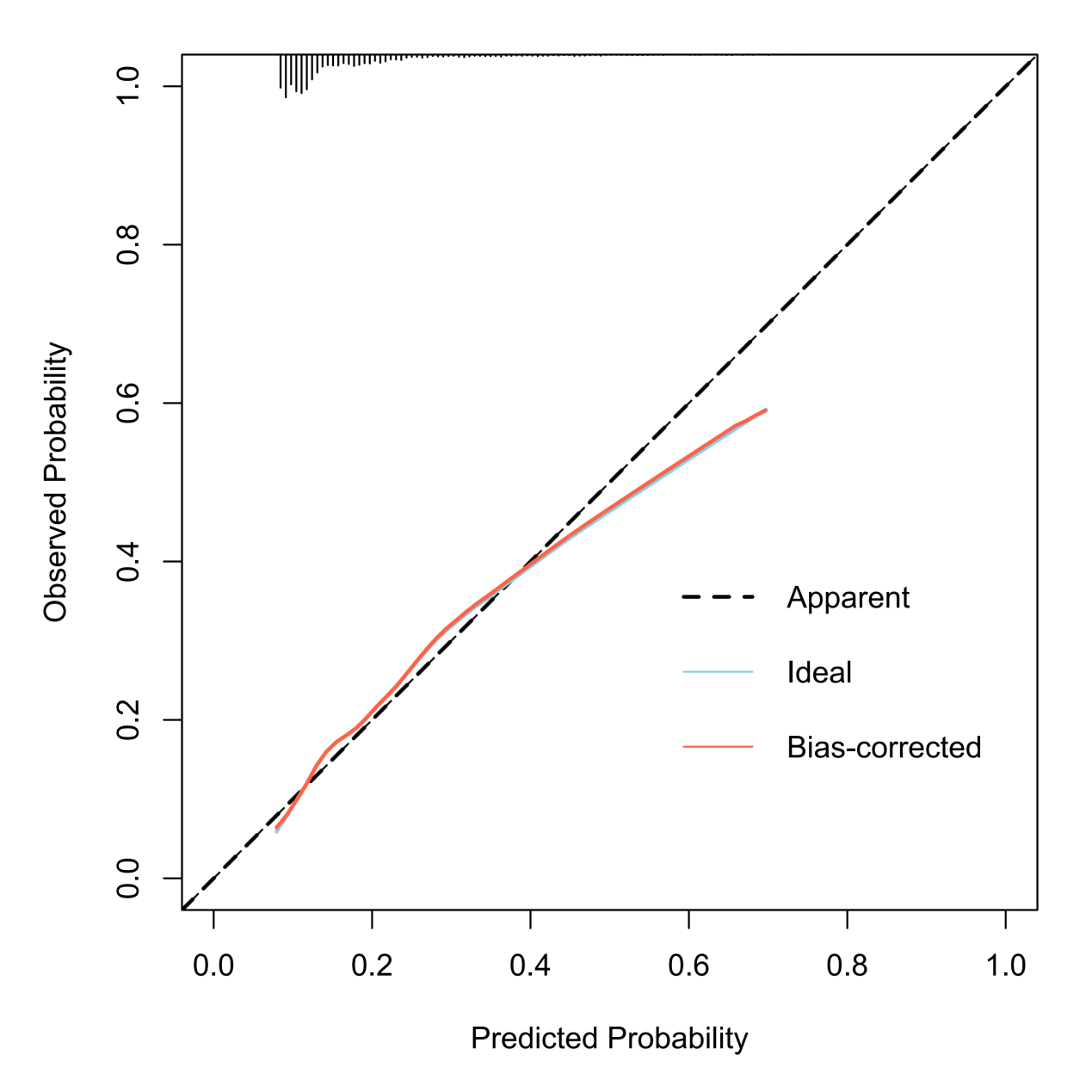
**

**Figure S5 Calibration curve of the model in the external validation set**

**
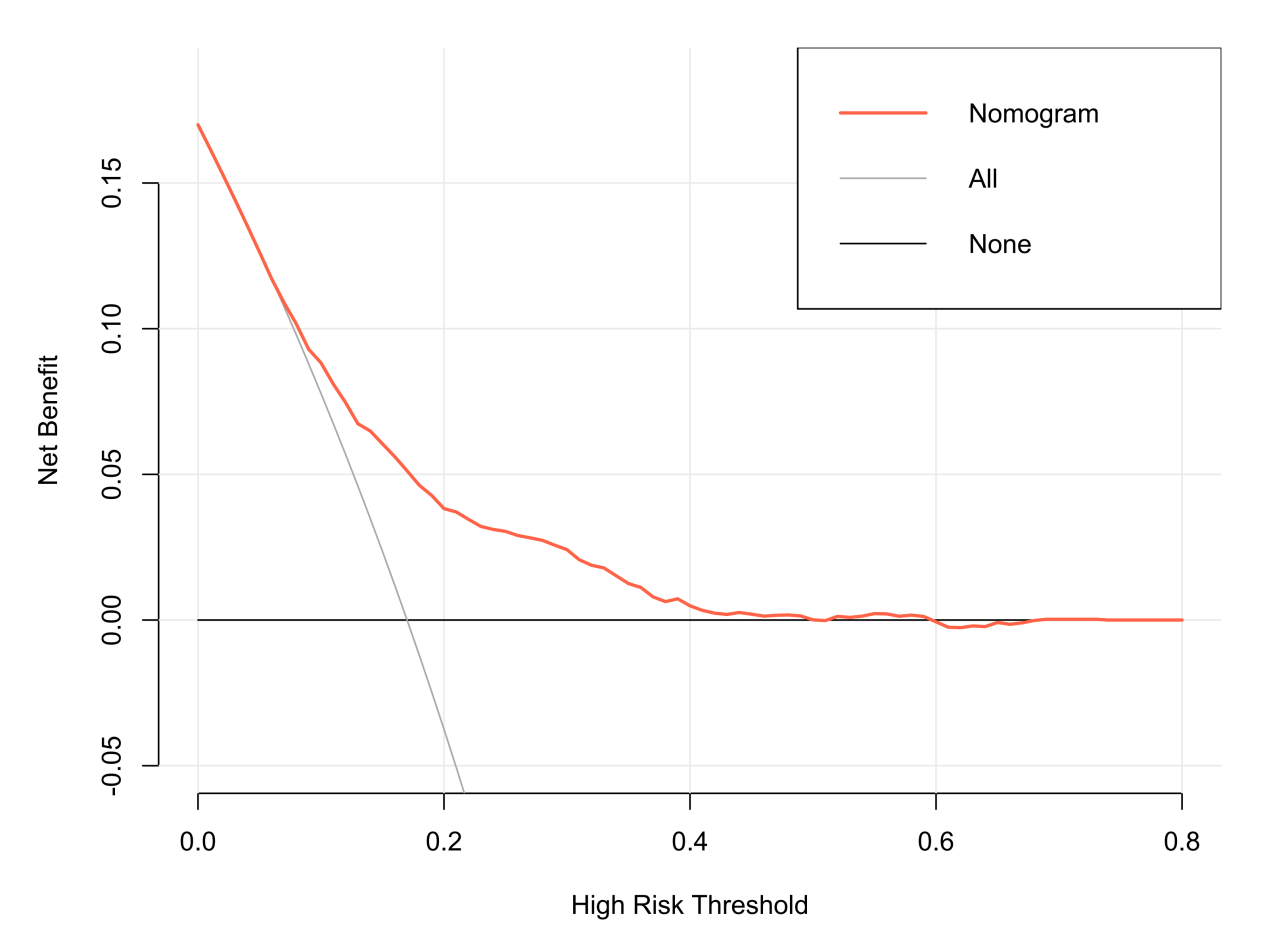
**

**Figure S6 DCA of the model in the internal validation set**

**
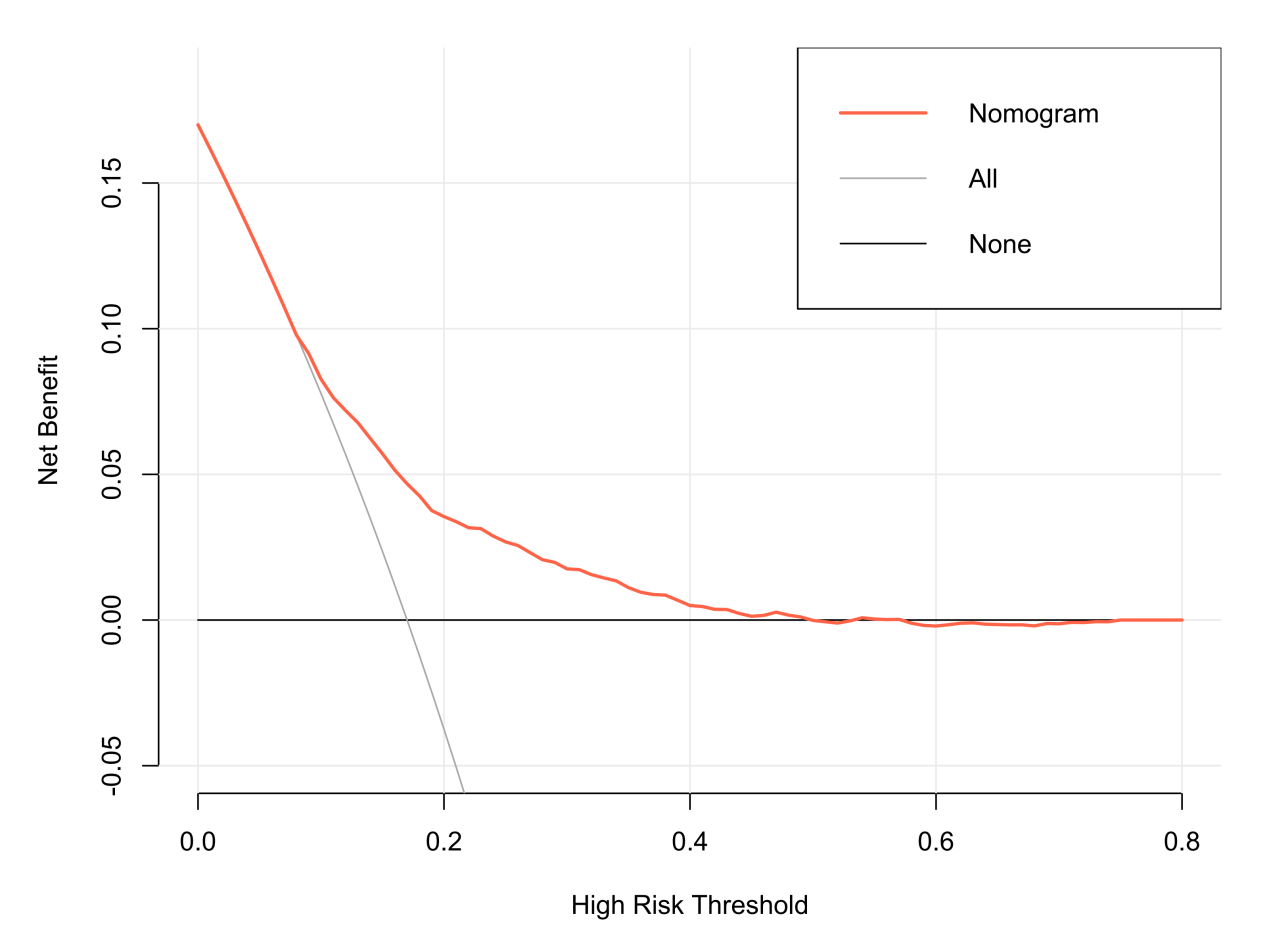
**

**Figure S7 DCA of the model in the external validation set**

**
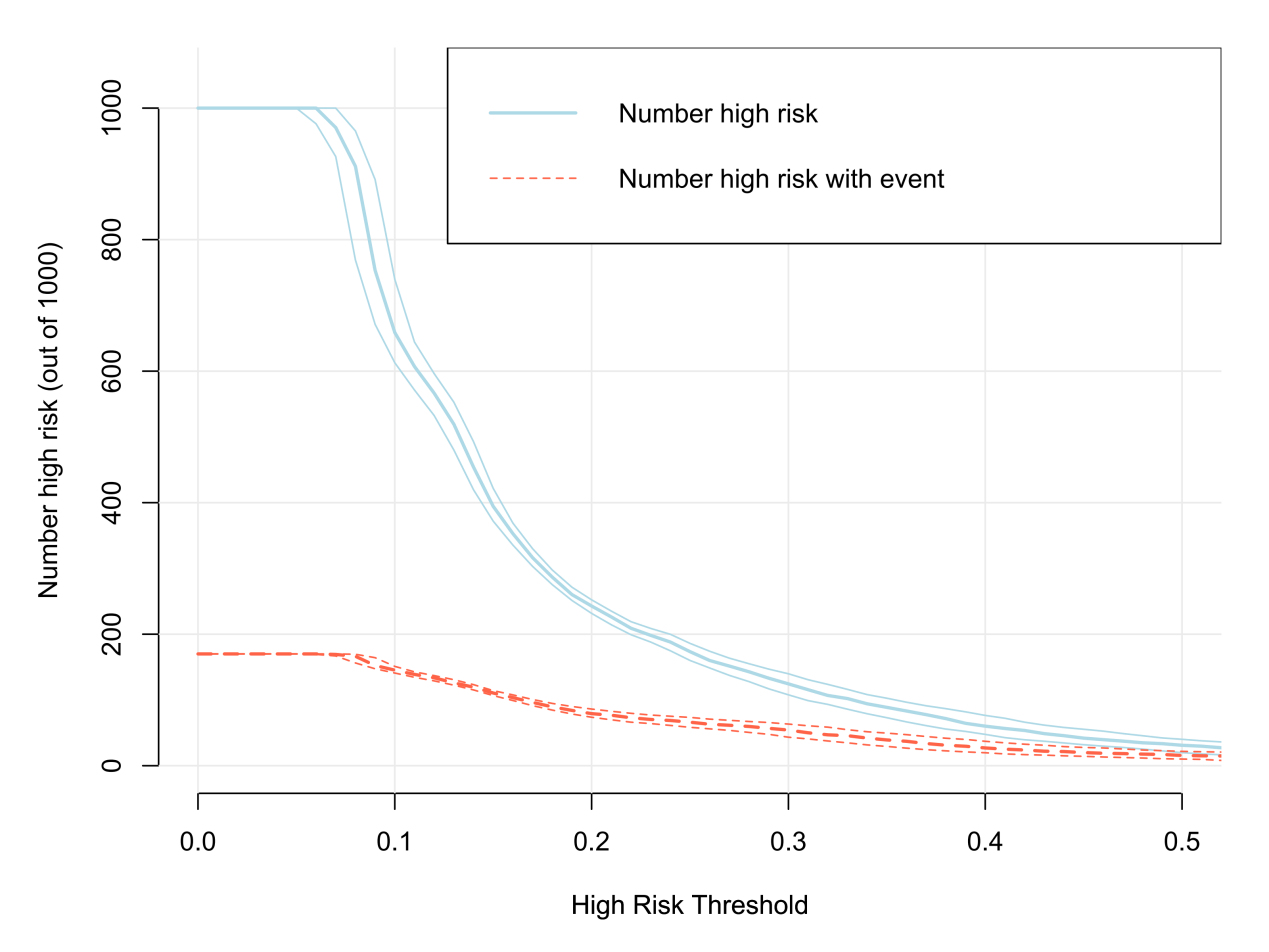
**

**Figure S8 CIC of the model in the internal validation set**

**
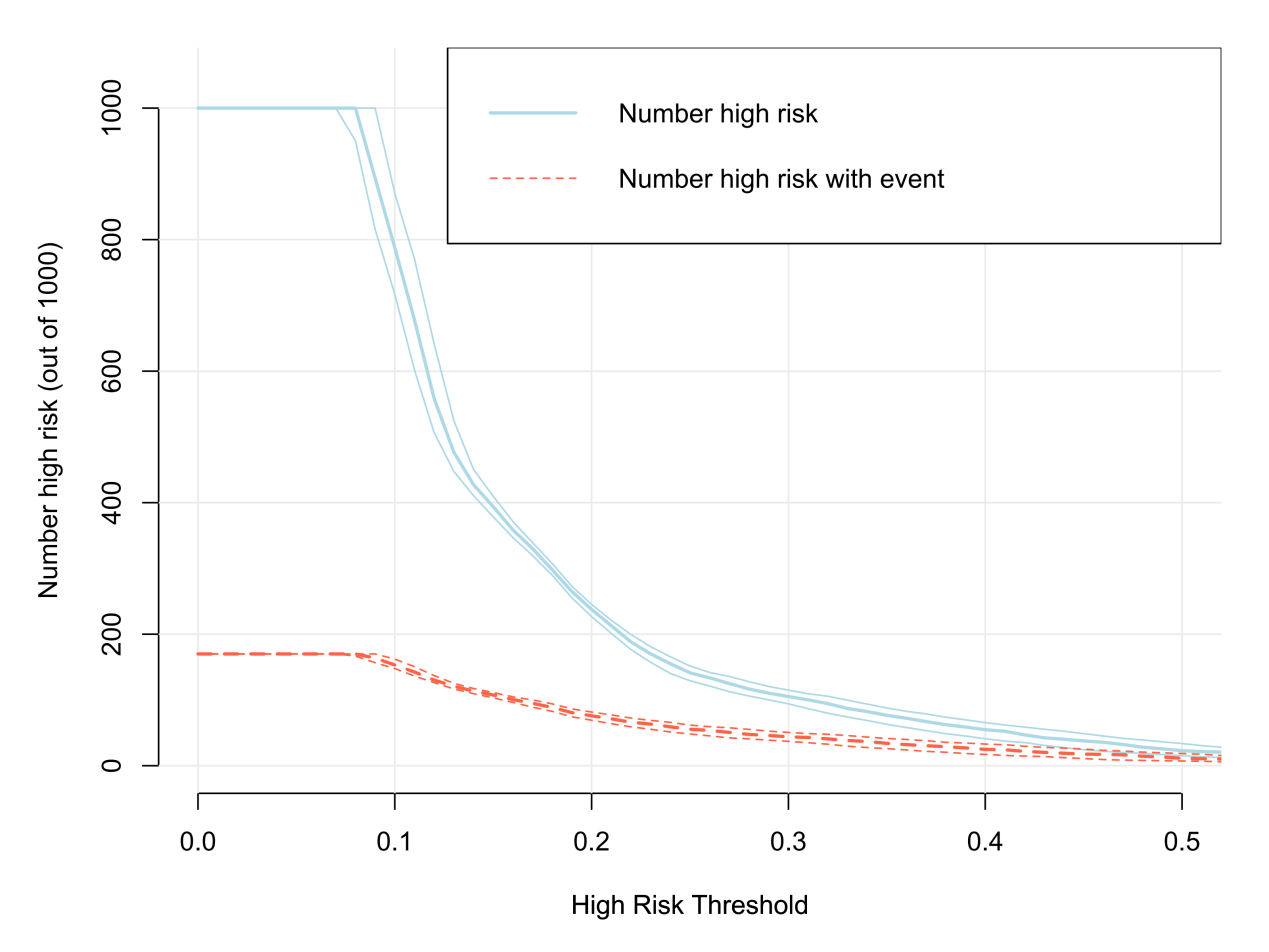
**

**Figure S9 CIC of the model in the external validation set**
